# EXPLORING HOW INTERPERSONAL FACTORS SHAPE HEALTH BEHAVIORS AMONG HISPANIC OLDER ADULTS: A SCOPING REVIEW

**DOI:** 10.1093/geroni/igae098.1102

**Published:** 2024-12-31

**Authors:** Barbara Mendez Campos, Karen Lyons, Bruna Lopez

**Affiliations:** Boston College, Chestnut Hill, Massachusetts, United States; Boston College, Chestnut Hill, Massachusetts, United States; Boston College, Chestnut Hill, Massachusetts, United States

## Abstract

As the U.S. experiences a significant demographic shift, particularly among older adults, this scoping review focuses on the Hispanic population, one of the largest segments of historically underrepresented racial/ethnic groups in this age cohort. An in-depth exploration of health behaviors becomes imperative with this demographic surge and an accompanying higher chronic illness profile. A noteworthy gap exists in understanding the influence of interpersonal factors on health behaviors, particularly among older Hispanic adults. This review systematically examines the existing literature on the roles of interpersonal factors within family and friends in shaping health behaviors among Hispanic older adults. Beyond the conventional categorization of cultural values like familismo, acculturation, fatalism, marianismo, machismo, collectivism, respeto, and religious beliefs as intrapersonal influences, the study emphasizes unraveling these factors within their relational context. It acknowledges the heterogeneity in adherence to these values and assesses their subsequent impact on health behaviors. Using the Joanna Briggs Institute (JBI) framework, the review identifies 39 relevant articles from six distinct databases. The findings underscore the urgency for future research utilizing mixed methods, multi-source, dyadic, and longitudinal approaches. Affirming the central role of interpersonal factors, predominantly involving daughters and wives, the study signals a gendered lens in relational dynamics within this community. Uncovering the roles of interpersonal factors significantly influencing various health behaviors, including physician visits, treatment choices, medication adherence, healthcare service use, and vaccinations, the review highlights the need for tailored interventions and further nuanced research in this critical area.

